# Determination of an optimally sensitive and specific chemical exchange saturation transfer MRI quantification metric in relevant biological phantoms

**DOI:** 10.1002/nbm.3614

**Published:** 2016-09-30

**Authors:** Kevin J. Ray, James R. Larkin, Yee K. Tee, Alexandre A. Khrapitchev, Gogulan Karunanithy, Michael Barber, Andrew J. Baldwin, Michael A. Chappell, Nicola R. Sibson

**Affiliations:** ^1^ Cancer Research UK and Medical Research Council Oxford Institute for Radiation Oncology, Department of OncologyUniversity of OxfordOxfordOX3 7LEUK; ^2^ Department of Mechatronics and Biomedical Engineering, Lee Kong Chian Faculty of Engineering and ScienceUniversiti Tunku Abdul RahmanMalaysia; ^3^ Physical and Theoretical ChemistryUniversity of OxfordOxfordOX1 3QZUK; ^4^ Institute for Biomedical EngineeringUniversity of OxfordOxfordOX3 7LEUK

**Keywords:** brain, CEST, metastases, MRI, pH

## Abstract

The purpose of this study was to develop realistic phantom models of the intracellular environment of metastatic breast tumour and naïve brain, and using these models determine an analysis metric for quantification of CEST MRI data that is sensitive to only labile proton exchange rate and concentration. The ability of the optimal metric to quantify pH differences in the phantoms was also evaluated.

Novel phantom models were produced, by adding perchloric acid extracts of either metastatic mouse breast carcinoma cells or healthy mouse brain to bovine serum albumin. The phantom model was validated using ^1^H NMR spectroscopy, then utilized to determine the sensitivity of CEST MRI to changes in pH, labile proton concentration, *T*_1_ time and *T*_2_ time; six different CEST MRI analysis metrics (MTR_asym_, APT*, MTR_Rex_, AREX and CESTR* with and without *T*_1_/*T*_2_ compensation) were compared.

The new phantom models were highly representative of the *in vivo* intracellular environment of both tumour and brain tissue. Of the analysis methods compared, CESTR* with *T*_1_ and *T*_2_ time compensation was optimally specific to changes in the CEST effect (i.e. minimal contamination from *T*_1_ or *T*_2_ variation). In phantoms with identical protein concentrations, pH differences between phantoms could be quantified with a mean accuracy of 0.6 pH units.

We propose that CESTR* with *T*_1_ and *T*_2_ time compensation is the optimal analysis method for these phantoms. Analysis of CEST MRI data with *T*_1_/*T*_2_ time compensated CESTR* is reproducible between phantoms, and its application *in vivo* may resolve the intracellular alkalosis associated with breast cancer brain metastases without the need for exogenous contrast agents.

**Abbreviations used**
APT*: three‐offset amide proton transfer metric
AREX: apparent relaxation due to exchange metric
BayCEST: Bayesian chemical exchange saturation fitting algorithm
BSA: bovine serum albumin
CEST: chemical exchange saturation transfer
CESTR*: chemical exchange saturation transfer ratio metric
EPI: echo planar imaging
MTR_asym_: magnetisation transfer asymmetry metric
MTR_Rex_: magnetisation transfer ratio relaxation due to exchange metric
NOE: nuclear Overhauser enhancement
PCA: perchloric acid
ppm: parts per million
qCEST: quantitative CEST

## 1 INTRODUCTION

Chemical exchange saturation transfer (CEST) is an MRI contrast mechanism that measures changes in signal from water protons owing to their association with other biomolecules and metabolites, particularly via exchange of protons with hydrolysable functional groups such as amides and amines.^1^, ^2^ CEST MRI has the potential to make non‐invasive measurements of pH,^3^ inform on areas of infiltrating tumour,^4^ differentiate tumour from radiation necrosis,^5^ and provide information concerning the conformation of proteins.^6^ Since the pH of tissues is highly regulated, and numerous pathologies interfere with this regulation, the application of CEST MRI to generate pH maps *in vivo* has received significant research interest recently.^7^

There is a clear need to develop a reliable, non‐invasive method of measuring tumour pH *in vivo*. One hallmark of cancerous tumours is their dysfunctional regulation of pH,^8^ leading to an acidotic extracellular space and slight alkalosis in the intracellular space.^9^ This change in pH has consequences for the efficacy of various treatments for tumours, and measurement of tumour pH may be useful in stratification of patients based on their expected responsiveness to particular therapies. In particular, brain metastasis, or secondary tumour spread to the brain, represents a major clinical problem, with poor prognosis and few therapeutic options. The development of better methods for interrogating the tumour microenvironment and targeting therapy may greatly enhance our ability to treat these tumours.

It is widely recognized that CEST is sensitive to more than just pH, which has led to various studies offering different explanations for the source of CEST contrast seen in tumours. Some studies claim that an increased protein concentration in tumour cells generates contrast between tumour and surrounding tissue.^10^, ^11^ However, others have measured insignificantly different protein concentration between rodent brain and implanted tumour, and suggest that the contrast is a result of *T*_1_ contamination of the signal.^12^ In addition, evidence suggests that the pH and labile proton concentration are difficult to separate from CEST measurements.^13^ Despite this difficulty, a metric that is not contaminated by relaxation time changes and only depends on the relevant physiological parameters is needed before the potential of using CEST MRI for pH measurement in tumours can be assessed.

To develop such a metric, previous studies have used simple phantoms with a single exchanging pool of protons.^13^, ^14^ These phantoms allow useful insights into the CEST MRI signal source, but do not adequately represent the complex *in vivo* intracellular biomolecular environment. Methods such as quantitative CEST (qCEST)^14^ and the Omega plot^15^ have been used in these phantoms to successfully quantify labile proton exchange rate and concentration independently. However, these methods require many *Z* spectra to be acquired with varying saturation parameters, which is impractical in a clinical environment. The apparent relaxation due to exchange (AREX) metric has also been proposed to correct for contamination of CEST effects by *T*_1_ relaxation of water,^12^, ^16^, ^17^, ^18^ but this method has not been validated in physiologically relevant phantoms.

The aims of this study, therefore, were the following: (i) to develop novel phantom models from cellular extracts that are representative of the *in vivo* intracellular environment of both normal brain and brain metastases; (ii) to use these phantoms to determine the sensitivity of the CEST MRI signal as measured by a number of different analysis metrics to changes in pH, labile proton concentration, *T*_1_ and *T*_2_; (iii) to identify an optimally specific analysis metric for quantification of CEST MRI data and (iv) to evaluate the ability of the optimal metric to quantify pH differences in these phantoms.

## 2 METHODS

### 2.1 Phantom preparation

Phantoms were prepared to represent the intracellular environment of naïve mouse brain and *in vitro* cultured 4T1‐GFP mouse metastatic breast carcinoma cells. The 4T1‐GFP cell line is commonly used as a mouse model of metastatic breast cancer, including studies of metastatic spread to the brain.^19^ It is an appropriate tumour model here as previous measurements of human breast cancers have displayed intracellular alkalosis.^20^ For the mouse brain phantoms, female BALB/c mice aged 6–8 weeks (*n* = 6) were terminally anaesthetized with sodium pentobarbital and transcardially perfused with 20 mL heparinized saline. Subsequently, the brains were removed, frozen in liquid nitrogen and stored at −80°C until further use. For the 4T1‐GFP cell phantoms, cells were cultured in Dulbecco's modified Eagle medium and passaged every second day to grow a sufficient number of cells.

Perchloric acid (PCA) extracts of either naïve brain or 4T1‐GFP cells were prepared. Briefly, either naïve mouse brains (*n* = 6) or a pellet of 4T1‐GFP cells (8 g) were homogenized and coated with 0.1 M HCl in methanol in a dry ice bath. Subsequently, the mixture was warmed to wet ice bath temperature, 0.02 M HCl and 3 M PCA added to the homogenizer and the precipitated protein sedimented by centrifugation (4800 *g*, 20 min, 4°C). The supernatant was neutralized to pH ~ 7 with KOH and the precipitated potassium perchlorate sedimented by centrifugation (4800 *g*, 20 min, 4°C). The supernatant was lyophilized. All volumes of solutions added were scaled to the weight of starting material to match metabolite concentration to their *in vivo*/intracellular values.

The lyophilized samples were divided into 26 equal fractions and added to bovine serum albumin (BSA) to produce phantoms representative of the intracellular environment of either naïve brain or 4T1‐GFP tumour cells. The pH (6.0–7.6, *n* = 6), BSA content (4–16% *w*/*v*, *n* = 7), *T*_1_ time (0.3–1.7 s, *n* = 7) and *T*_2_ time (29–140 ms, *n* = 6) of each phantom was serially varied for a total of 52 phantoms (*n* = 26 for each cell type). *T*_1_ and *T*_2_ relaxation times were varied by addition of gadolinium‐DTPA (Omniscan, GE Healthcare) and iron nanoparticles (25–30 nm diameter), respectively. Where pH and BSA content were not varied, the phantoms were pH 7.4 with a BSA concentration of 8% *w*/*v*. BSA has been used in previous studies as a protein representative of the *in vivo* protein pool, and 8% w/v is a reasonable assumption of the protein content of the rodent brain.^21^, ^22^ The BSA was not cross‐linked to avoid macromolecular magnetization transfer effects being introduced, and the phantom pH was titrated after addition of BSA.

### 2.2 Validation of tissue/cell extract phantoms

The validity of the PCA extract supplemented with BSA model as a reasonable representation of the intracellular environment of brain/tumour cells *in vivo* was confirmed using high‐resolution NMR spectroscopy. One‐dimensional ^1^H spectra with WATERGATE solvent suppression were acquired (see Section 2.3) from samples of PCA‐extracted naïve mouse brain, *in vitro* cultured 4T1‐GFP cells, and subcutaneous 4T1‐GFP tumours. The subcutaneous tumours were grown by injecting 5 × 10^5^ 4T1‐GFP cells in 100 μL PBS subcutaneously into female BALB/c mice aged 6–8 weeks (*n* = 6). Tumours were allowed to grow until 10 mm geometric mean diameter, at which point the mice were terminally anaesthetized with sodium pentobarbital and transcardially perfused with 20 mL heparinized saline. The tumours were then isolated from the surrounding skin and fat, frozen in liquid nitrogen and stored at −80°C until further use.

Additional spectra were obtained from the lysate of *in vitro* cultured 4T1‐GFP cells and the respective phantom model (PCA‐extracted *in vitro* cultured 4T1‐GFP cells supplemented with 8% *w*/*v* BSA). For the PCA extracts, the lyophilized samples were dissolved in pH 7.4 potassium phosphate buffer (1 M) to provide suitable buffering capability over the range required for this study. The cell lysate sample was produced by suspending 4T1‐GFP cells in NP‐40 lysis buffer (2.74 mL 1 M NaCl, 2 mL 200 mM pH 7 Tris HCl, 80 μL 0.5 M EDTA, 200 μL NP‐40, 4.98 mL dH_2_O) and sedimenting the precipitated cellular membranes by centrifugation (21 000 *g*, 20 min, 4°C). All samples were prepared to a final volume of 600 μL with 5% D_2_O.

In addition, samples of 8% *w*/*v* BSA in potassium phosphate buffer (1 M, pH 7.4) were supplemented with varying concentrations of PCA‐extracted *in vitro* cultured 4T1‐GFP cells (1×, 1.5× and 2× metabolite concentration) to confirm that the presence of metabolites from the PCA‐extracted cells in the sample influences the measured CEST spectrum.

### 2.3 Solution NMR experiments

Proton spectra were acquired using a vertical bore 600 MHz (14.1 T) spectrometer (Agilent Technologies, Santa Clara, CA, USA) using a WATERGATE sequence with a relaxation delay of 2 s, an acquisition time of 2 s and 128 transients per free induction decay recorded. The carrier was centred on water (4.7 ppm) with a sweep width of 9551 Hz. Spectra were processed using NMRPipe.^23^

### 2.4 MRI experiments

All MRI experiments were performed using a horizontal bore 400 MHz (9.4 T) spectrometer (Agilent Technologies) with a volume transmit–receive coil (internal diameter 40 mm, RAPID Biomedical, Rimpar, Germany). Shimming was performed prior to each experiment to minimize the *B*_0_ field inhomogeneity. CEST images were acquired of 26 phantoms simultaneously using a saturation scheme of 300 Gaussian pulses of 26 ms duration and 180° flip angle each (50% duty cycle, equivalent continuous wave saturation power 0.8 μT) at 85 saturation frequencies spaced equally between ±10 ppm, followed by an eight‐shot spin‐echo echo planar imaging (EPI) readout. Additional images were acquired following saturation at ±100 ppm for normalization; field of view 38 mm × 38 mm, matrix size 32 × 32, slice thickness 2 mm, echo time (*T*_E_) 8.22 ms and repetition time (*T*_R_) 7.85 s. Total scan time for each set of phantoms was 3 h 6 min.

In addition to CEST imaging, the *T*_1_ and *T*_2_ relaxation times of each phantom were measured using inversion recovery (*T*_R_ = 10 s, *T*_E_ = 8.22 ms, inversion time (*T*_I_) varied in nine steps from 13.14 ms to 8 s, signals fitted to *M*_*z*_ = *M*_0_(1–2 exp(−*T*_I_/*T*_1_)) and spin echo (*T*_R_ = 10 s, *T*_E_ varied in 10 steps from 30 ms to 160 ms, signals fitted to *M*_*z*_ = *M*_0_ exp(−*T*_E_/*T*_2_) experiments, respectively. In both cases eight‐shot spin‐echo EPI readout was used to acquire images.

### 2.5 MRI data processing

All MRI data were processed in MATLAB (MathWorks, Natick, MA, USA). The relaxation maps of the water pool were obtained by least square fitting of the measured intensity against the inversion time (*T*_1_ map) and echo time (*T*_2_ map). Six metrics were used to analyse the *Z* spectra: conventional asymmetry analysis (MTR_asym_),^1^ multiple‐offset analysis (APT*),^24^ inverse *Z*‐spectrum multiple‐offset analysis (MTR_Rex_),^12^ AREX,^12^ and two variants of a Bayesian model‐based analysis (CESTR*),^13^ as defined in Equations (1)–5, respectively. *B*_0_ inhomogeneity was corrected prior to MTR_asym_, APT* and MTR_Rex_ analysis by shifting the minimum point of the *Z* spectrum to 0 ppm on a voxel‐wise basis; CESTR* corrects for *B*_0_ inhomogeneity during the analysis. All analyses were performed on a voxel‐wise basis, and the data presented for each phantom are the mean ± standard deviation for a fixed‐area region of interest (ROI) over each phantom.

(1) $${\mathrm{MTR}}_{\text{asym}}\left(\omega \right)=\frac{Z\left(-\omega \right)-Z\left(\omega \right)}{Z_{0}}$$

(2) $${\mathrm{APT}}^{*}\left(\omega \right)=Z_{\mathrm{ref}}\left(\omega \right)-Z\left(\omega \right)$$

(3) $${\mathrm{MTR}}_{\mathrm{Rex}}\left(\omega \right)=\frac{1}{Z\left(\omega \right)}-\frac{1}{Z_{\mathrm{ref}}\left(\omega \right)}$$

(4) AREXω=MTRRexω/T1

(5) $${\text{CESTR}}^{*}\left(\omega \right)=\frac{\left[S_{1-\text{pool}}\left(\omega \right)-S_{2-\text{pool}}\left(\omega \right)\right]}{Z_{0}}$$

In Equations (1)–5, *ω* is the offset frequency of interest, *Z*(*ω*) is the signal measured following saturation at the frequency *ω*, *Z*_0_ is the signal measured following saturation at ±100 ppm, $Z_{\mathrm{ref}}\left(\omega \right)=\frac{Z\left(\omega +\delta \omega \right)-Z\left(\omega -\delta \omega \right)}{2}$, and *S*_*n* − pool_ is the signal from a simulated Z spectrum with *n* pools, and labile pool properties defined by those measured by a Bayesian model‐based algorithm,^13^ implemented in BayCEST as part of the FMRIB Software Library (www.fmrib.ox.ac.uk/fsl/baycest). CEST effects were measured for all metrics at *ω* = 2.8 ppm with δ*ω* = 1.4 ppm.

Three pools were fitted using BayCEST: the water pool at 0 ppm (W), the labile amine proton pool at 2.8 ppm (CEST) and an exchange pool centred at −3.5 ppm. BayCEST fits the Bloch–McConnell equations to the measured *Z* spectra with the exchange rate and relaxation times for each pool defined by Bayesian prior distributions. BayCEST measures fitted values of the exchange rate and relative concentration of the protons in each pool, and *T*_1_ and *T*_2_ relaxation times for all proton pools (Supplementary Figure S1). BayCEST was run for each phantom with ‘default’ values for the mean of the prior distributions for water *T*_1_ (1.8 s) and *T*_2_ (100 ms). Subsequently, the water *T*_1_ and *T*_2_ times measured from each phantom in the *T*_1_ and *T*_2_ maps were included as the means of the associated prior distributions in the model fitting (Supplementary Table S1). In both cases the *T*_1_ and *T*_2_ values remained as parameters within the fitting procedure to be estimated from the data.

CESTR* was calculated from the 2.8 ppm pool by simulating one‐pool (W only) and two‐pool (W + CEST) systems using only the fitted estimates of exchange rate and relative concentration, and measuring the difference in signal at 2.8 ppm (Supplementary Figure S2). This produced two values for CESTR*—one calculated with ‘default’ priors for water *T*_1_ (1.8 s) and *T*_2_ (100 ms), and a second with phantom specific measured *T*_1_ and *T*_2_ prior means—hereafter called ‘CESTR* with measured *T*_1_/*T*_2_ priors’. The fitted exchange rate was not used directly, as a degree of correlation exists between the fitted exchange rate and concentration.^13^ Further details of the fitting and analysis procedure for CESTR* can be found in the Supplementary Methods.

### 2.6 Determination of optimal metric

The relationship between the calculated CEST effect using each metric and the serially varied parameters was determined by linear regression, and its absolute value (in |% *M*_0_|/parameter unit change) compared between phantom models using a *t* test corrected for multiple comparisons using the Holm–Sidak method.^25^ Statistical significance was defined as *P* < 0.05, which after correction gave an effective significance level of *P* < 0.003. These comparisons were used to determine whether pooling the data from the two phantom models was appropriate.

Subsequently, a further multi‐parameter linear regression model was used to determine the optimal analysis metric in terms of specificity. Data from both phantom models (tumour and naïve brain) were combined and fitted to a model of the form of Equation (6), which describes the response of an ideal CEST quantification metric (i.e. only dependent on pH and [BSA]).

(6) $$\text{CEST effect}\;\left(\%M_{0}\;\right)=\alpha \;\mathrm{pH}+\beta \left[\mathrm{BSA}\right]+\varepsilon .$$

The coefficients *α* , β , ε of Equation (6) are constants $\left(\mathrm{units}\left[\alpha \right]=,\frac{\left(\%,M_{0}\right)}{\mathrm{pH}},\left[\beta \right],=,\frac{\left(\%,M_{0}\right)}{\left(\%,\mathrm{BSA}\right)},\left[\varepsilon \right]=\%M_{0}\right)$ that describe the level of sensitivity of the experimentally measured data to the varied parameters in the phantoms. Using these coefficient values and the known pH and [BSA] for each phantom, theoretical CEST effects were calculated and correlated to the experimentally measured CEST effects for each metric. The *R* ^2^ values for these correlations were used as indications of the specificity of the metric to changes in only pH and [BSA]. The optimal metric was defined as that with the highest *R* ^2^ value for the correlation between calculated and experimentally measured CEST effects, which represents a metric with minimal contamination by *T*_1_ and *T*_2_ time.

### 2.7 Measurement of pH differences using optimal metric

For the optimal metric, differences in experimentally measured CEST effects were calculated between each pair of phantoms (*Δ*CEST = CEST1 − CEST2). These differences were tested for statistical significance, defined as a difference larger than the standard deviations on each CEST effect measurement added in quadrature $\left(\text{ΔCEST}>\sigma_{\text{ΔCEST}}=\sqrt{{\sigma_{\text{CEST}1}}^{2}+{\sigma_{\text{CEST}2}}^{2}}\right)$.

For statistically significant differences in CEST effect, pH differences as measured by pH probe (‘Experimental ΔpH’) and back calculated from the linear regression coefficients from Equation (6) (‘Calculated ΔpH’) were correlated to verify the suitability of the metric for measuring pH differences.

## 3 RESULTS

### 3.1 Phantom validation

**Figure 1:**
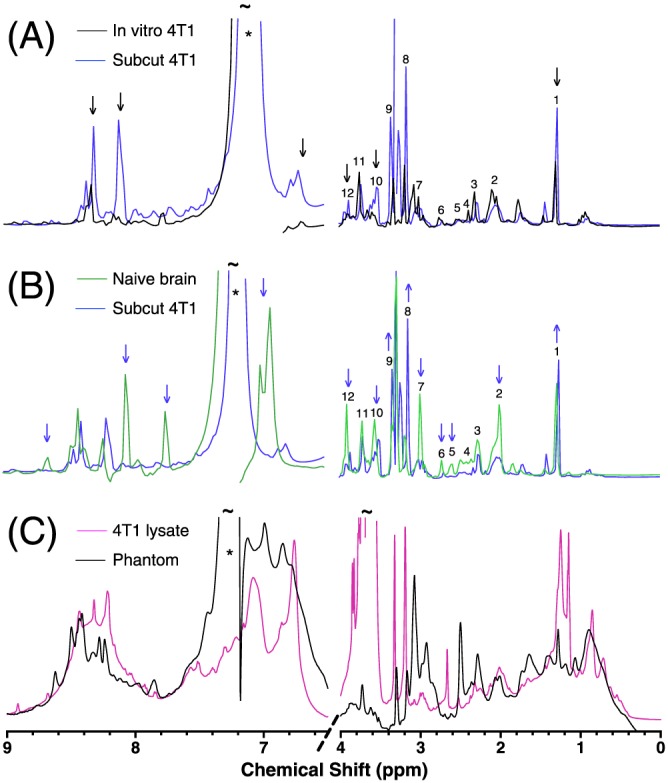
One‐dimensional ^1^H NMR spectra, showing (A), the metabolic similarity of 4T1‐GFP cells grown *in vivo* as a subcutaneous tumour (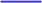) or *in vitro* (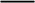) in culture flasks, (B), the metabolic distinctness of 4T1‐GFP mouse mammary carcinoma cells (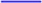) from naïve mouse brain (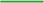) and (C), the similarity of the broad line shape component of the spectrum from cell lysate of 4T1‐GFP cells (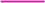) to the PCA extract of the same cell line supplemented with 8% w/v BSA (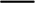). Coloured arrows signify relative differences between spectra, and * signifies signal from a contaminant from the PCA extraction procedure. ~ signifies points where the peaks exceed the axis limits. Numbers are metabolite identification: 1, Lac; 2, NAA; 3, Glu; 4, Gln; 5, NAA; 6, Asp; 7, Cr/PCr; 8, PC/GPC; 9, Tau; 10, Myo‐ins; 11, Asc/Gln/Glu/GSH; 12, Cr/PCr

NMR spectra of the tissue and cellular models were compared. The metabolite peaks in spectra obtained from PCA‐extracted *in vitro* cultured 4T1‐GFP cells and the same cells grown *in vivo* as a subcutaneous tumour were very similar in magnitude (Figure 1A). Thus, *in vitro* cultured 4T1‐GFP cells are metabolically similar to an *in vivo* tumour. However, clear differences were seen in the distribution and magnitude of metabolite peaks from PCA‐extracted naïve mouse brain and *in vivo* tumour, revealing their distinctly different metabolite compositions (Figure 1B). Upon comparing the spectra from the lysate of *in vitro* cultured 4T1‐GFP cells and the representative phantom model (Figure 1C), the marked similarities in the magnitude of the broad protein lineshapes indicate that the phantom (extract) model is a reasonable approximation of the intracellular environment of 4T1‐GFP cells.

**Figure 2:**
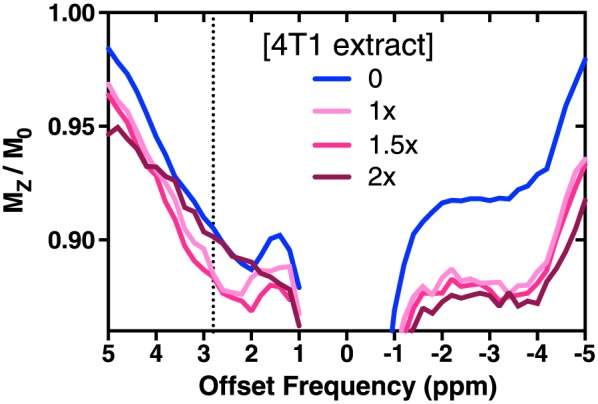
*Z* spectra of 8% *w*/*v* BSA phantoms at pH 7.2 supplemented with PCA extract from 4T1‐GFP cells, replicating metabolite concentrations of 1×, 1.5× or 2× intracellular levels. The 4T1‐GFP intracellular metabolites clearly alter the measured *Z* spectrum from the BSA‐only *Z* spectrum (0×). The CEST effect at 2.8 ppm broadens with increasing metabolite content, consistent with an increasing average chemical exchange rate

Clear differences between the *Z* spectra from phantoms of BSA only and those containing 4T1‐GFP cell PCA extracts were evident in the −1.0 to −5.0 ppm region, and indicates nuclear Overhauser enhancement (NOE)‐mediated saturation of the water signal owing to the presence of the intracellular metabolites (Figure 2). The CEST effect centred at 2.8 ppm is also altered with increasing concentration of PCA‐extracted tissue.

### 3.2 Quantification of CEST effects by multiple metrics

**Figure 3:**
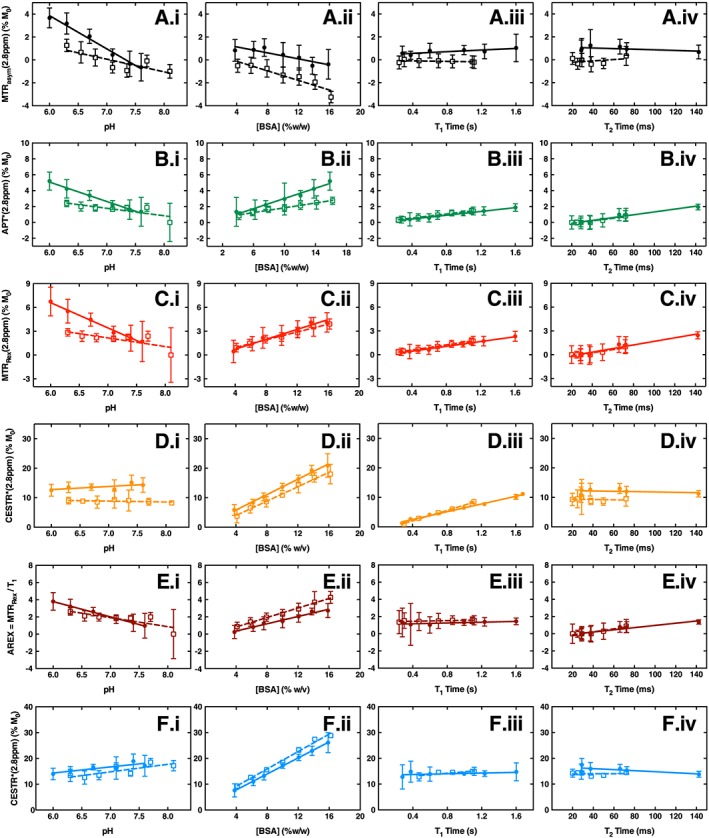
Linear regression relationships of MTR_asym_ A, APT* B, MTR_Rex_ C, CESTR* calculated with default *T*_1_/*T*_2_ priors D, AREX E, and CESTR* calculated with measured *T*_1_/*T*_2_ priors F, as a function of pH (i), BSA content (ii), *T*_1_ time (iii) and *T*_2_ time (iv) for the CEST effect at 2.8 ppm in phantoms containing 8% w/v BSA and PCA extract from 4T1‐GFP cells (solid circles) and naïve mouse brains (open squares). Solid and dashed lines are the fitted linear regression relationships to tumour and naïve brain phantoms, respectively

The relationships between the measured CEST effect at 2.8 ppm and the pH, BSA concentration, *T*_1_ time and *T*_2_ time were determined using multiple analysis metrics for phantoms containing BSA supplemented with extracts from either 4T1‐GFP cells or naïve mouse brain (Figure 3). The *T*_2_ time was considered constant for increasing concentrations of gadolinium‐DTPA, and the *T*_1_ time constant for increasing concentrations of iron nanoparticles, as these varied minimally compared with the objective relaxation time (Supplementary Figure S3).

Significance levels for all linear regression relationships are shown in Table 1. Significant decreases in MTR_asym_, APT* and MTR_Rex_ were observed with increasing pH, which is expected for the fast‐exchanging amine protons at this frequency^21^ (Figure 3A.i–C.i). Conversely, however, since CESTR* estimates the exchange rate and concentration directly from the *Z* spectrum, an increase in CESTR* (calculated with default *T*_1_/*T*_2_ priors) was seen as pH increased (Figure 3D.i). The AREX metric also showed a significant decrease as pH increased (Figure 3E.i), and the increase in CESTR* became significant when measured *T*_1_/*T*_2_ priors were included (Figure 3F.i).

**Table 1:** *P* values for the linear regression fits of CEST effect as measured by the four analysis metrics compared, for each of the varied parameters (pH, [BSA], *T*_1_ and *T*_2_), in both phantom models. *P* values marked * are statistically significant results, defined as *P* < 0.05

		pH	[BSA]	*T*_1_ Time	*T*_2_ Time
4T1‐GFP cell phantoms	MTR_asym_	<0.0001*	0.006*	0.04*	0.25
	MTR_Rex_	<0.0001*	<0.0001*	<0.0001*	0.001*
	APT*	<0.0001*	0.0004*	<0.0001*	0.001*
	CESTR* (default *T*_1_/*T*_2_ priors)	0.12	<0.0001*	<0.0001*	0.56
	AREX	<0.0001*	<0.0001*	0.12	0.003*
	CESTR* (measured *T*_1_/*T*_2_ priors)	0.02*	<0.0001*	0.32	0.11
Naïve brain phantoms	MTR_asym_	0.02*	0.002*	0.33	0.46
	MTR_Rex_	0.05	<0.0001*	<0.0001*	0.006*
	APT*	0.04*	<0.0001*	<0.0001*	0.006*
	CESTR* (default *T*_1_/*T*_2_ priors)	0.243	<0.0001*	<0.0001*	0.72
	AREX	0.03*	<0.0001*	0.06	0.006*
	CESTR* (measured *T*_1_/*T*_2_ priors)	0.03*	<0.0001*	0.06	0.96

Significant increases in all metrics apart from MTR_asym_ were evident with increasing protein concentration (Figure 3B.ii–F.ii). In contrast, MTR_asym_ decreased as protein concentration increased (Figure 3A.ii). APT*, MTR_Rex_ and CESTR* (default *T*_1_/*T*_2_ priors) were found to be sensitive to changes in both proton relaxation times in both phantom models (Figure 3B.iii,iv–D.iii,iv), whilst MTR_asym_ was sensitive only to changes in *T*_1_ in the 4T1‐GFP phantom model (Figure 3A.iii,iv). Some measurements of MTR_Rex_ and APT* were negative because in these instances the relaxation time change resulted in no discernible CEST peak at 2.8 ppm (see Supplementary Figure S4). Since a negative CEST effect measurement is unphysical, these measurements were set to zero. As expected, AREX was insensitive to *T*_1_ relaxation time variations in both phantom models, but sensitive to *T*_2_ time changes (Figure 3E.iii,iv). CESTR* (measured *T*_1_/*T*_2_ priors) was the only metric insensitive to both relaxation times in both models (Figure 3F.iii,iv).

Significance levels for the comparisons of linear regression gradients between the two phantom models are shown in Table 2. Significant differences were found between phantom models for MTR_asym_, APT* and MTR_Rex_ as pH was varied, and for CESTR* with default *T*_1_/*T*_2_ priors as *T*_1_ time was varied (Figure 3A.i–C.i,D.iii, *P* values in Table 2).

**Table 2:** *P* values for the results of *t* tests comparing the linear regression gradient values for CEST effect measurements from 4T1‐GFP and naïve brain phantom. *P* < 0.05 was defined as statistically significant, with correction for multiple *t* tests using the Holm–Sidak method. *P* values marked * are statistically significant differences

	MTR_asym_	MTR_Rex_	APT*	CESTR* (default *T*_1_/*T*_2_ priors)	AREX	CESTR* (measured *T*_1_/*T*_2_ priors)
pH	0.001*	0.001*	0.002*	0.02	0.02	0.45
[BSA]	0.04	0.01	0.02	0.10	0.003	0.04
*T*_1_ time	0.05	0.10	0.06	0.001*	0.54	0.03
*T*_2_ time	0.49	0.36	0.36	0.99	0.09	0.16

### 3.3 Determination of optimal metric

MTR_asym_, APT*, MTR_Rex_ and CESTR* with default *T*_1_/*T*_2_ priors all showed a significant sensitivity to serial variation in *T*_1_ and *T*_2_ time, indicating the contaminant effect that the water relaxation times have on these metrics when quantifying CEST effects. Of the two methods that incorporate compensation for relaxation times (AREX and CESTR* with measured *T*_1_/*T*_2_ priors), the CESTR* measurements showed minimal sensitivity to serial variation in either *T*_1_ or *T*_2_ time (Figure 3F.iii,iv). As expected, AREX was not significantly sensitive to variations in *T*_1_ time, but did vary with *T*_2_ time. Notably, CESTR* with measured *T*_1_/*T*_2_ priors was the only metric to show no significant dependence on *T*_1_ or *T*_2_ time in either phantom model, indicating the specificity of CESTR* to measuring changes in only the CEST pool properties (Table 1).

**Figure 4:**
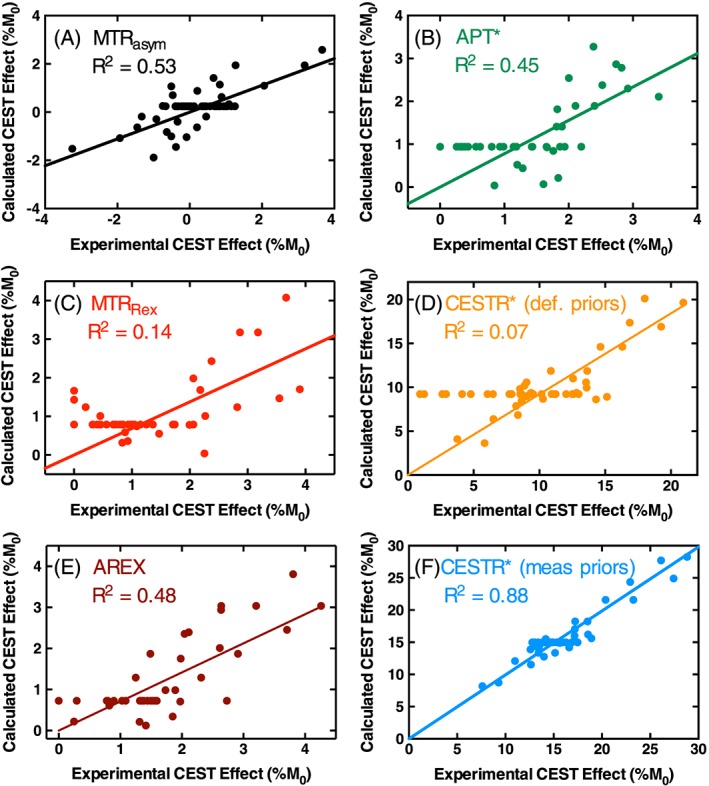
Correlations between calculated CEST effects from Equation (6) and the experimentally measured CEST effects in each phantom as measured by each analysis metric: MTR_asym_ A, APT* B, MTR_Rex_ C, CESTR* calculated with default *T*_1_/*T*_2_ priors D, AREX E, and CESTR* calculated with measured *T*_1_/*T*_2_ priors F. *R* ^2^ values are MTR_asym_ = 0.53, MTR_Rex_ = 0.14, APT* = 0.45, CESTR* with default priors =0.07, AREX =0.48 and CESTR* with measured priors =0.88

Correlation analysis between the experimentally measured CEST effects and the theoretically calculated CEST effects from Equation (6) yielded the highest *R* ^2^ value (0.88) for CESTR* with measured *T*_1_/*T*_2_ priors (Figure 4A–F). This high *R* ^2^ value means that CESTR* with measured *T*_1_/*T*_2_ priors is dependent only on pH and protein concentration. MTR_asym_, APT*, MTR_Rex_ and CESTR* with default *T*_1_/*T*_2_ priors (Figure 4A–D) display constant calculated CEST effect in many cases, because these metrics do not directly account for the effects of *T*_1_ or *T*_2_ variations, making it difficult to use these metrics for pH estimation. One contributing factor to the low *R* ^2^ values observed for MTR_asym_ (0.53), MTR_Rex_ (0.14) and APT* (0.45) may be the significant difference in linear regression gradients between tumour and naïve brain phantom models as pH is varied. However, it is unlikely that this is the only factor, since AREX and CESTR* with default *T*_1_/*T*_2_ priors both display similarly low *R* ^2^ values (0.48 and 0.07 respectively) with non‐significant differences between phantom models. On this basis, the optimal metric was found to be CESTR* with measured *T*_1_/*T*_2_ priors.

### 3.4 Measuring pH differences using CESTR*

**Figure 5:**
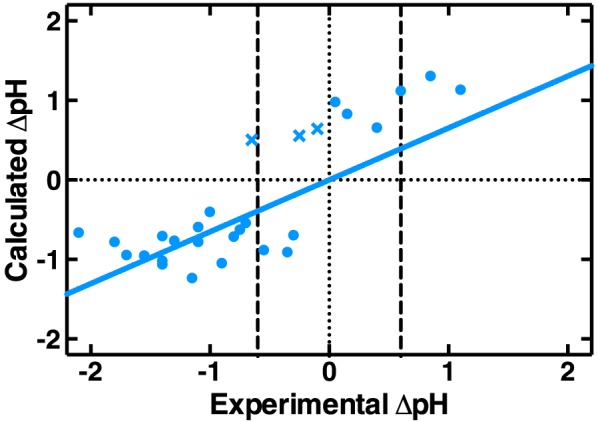
Correlation between ΔpH calculated using a rearrangement of Equation (6) for statistically significant ΔCESTR* measurements between phantoms, and the ΔpH experimentally measured by pH probe. The *R* ^2^ value is 0.61. Cross markers (**X**) indicate the data points where CESTR* incorrectly predicted the sign of the pH change

Differences in the experimentally measured CESTR* values (with measured *T*_1_/*T*_2_ priors) from each phantom were measured and tested for statistical significance. A total of 28 ΔCESTR* values were found to be significant. For these differences, the experimentally measured ΔpH and the calculated ΔpH using a rearrangement of Equation (6) were correlated (Figure 5). These findings validate CESTR* as a reliable measure of ΔpH by CEST MRI, with a root mean square deviation for the correlation in Figure 5—and expected quantitative pH accuracy—of 0.6 pH units for the case of no variation in protein concentration.

Further, considering the data points in distinct groups based on the magnitude of the experimental ΔpH value, CESTR* correctly predicted the sign of ΔpH in 94% of cases (16/17) where experimental ΔpH < −0.6 pH units and 100% of cases where experimental ΔpH > 0.6 pH units (3/3). For the group where experimental ΔpH was within ±0.6 pH units of zero, CESTR* was successful in 75% of cases (6/8).

## 4 DISCUSSION

The current study examines the sensitivity and specificity of various CEST MRI quantification methods, in novel phantom models that closely reflect the intracellular environment of brain metastases and naïve mouse brain. In contrast to all other metrics, CESTR* with measured *T*_1_/*T*_2_ priors was found to be sensitive only to variations in pH and [BSA] in both tumour and naïve brain phantom models. CESTR* can remove the effects of *T*_1_ and *T*_2_, which are known to vary with pathological changes *in vivo*,^26^ from the CEST measurement, allowing a more accurate quantification of the CEST effect. We propose, therefore, that CESTR* with measured *T*_1_ and *T*_2_ priors is the most specific metric for quantification of CEST MRI data. CESTR* with measured *T*_1_/*T*_2_ priors can be applied robustly both between samples, and between voxels within a single experiment, to measure pH differences with an accuracy of 0.6 pH units in these phantoms.

Other studies have suggested that the *T*_1_ contamination of the CEST signal is counteracted by changes in water content *in vivo*, and that the CEST contrast in tumours originates from a higher mobile protein concentration.^27^ If this is the case, it may be difficult to quantify pH in tumours as both the pH and protein concentration may be changing simultaneously. However, as shown by this study and others,^12^, ^28^ *T*_1_ correction is absolutely necessary for reliable, specific quantification of the CEST signal, regardless of the counteracting effect of water content. Assuming that the effects of *T*_1_ and water content perfectly cancel may lead to inaccurate quantification of CEST effects *in vivo* in pathologies where only one of these parameters changes.

MTR_asym_ measured from our phantoms decreased as the protein concentration increased, in contrast to previous studies.^5^, ^10^, ^21^ This discrepancy can be explained by the difference in saturation parameters used in the studies. In this study, a 7.8 s pulse train with CW equivalent power 0.8 μT was employed, which is preferentially sensitive to slowly exchanging protons. The effect of these saturation parameters is to enhance the NOE effects seen from the BSA and extracted metabolites in the phantoms. As the protein concentration increases, these NOE effects increase more than the CEST effects at 2.8 ppm, leading to a decreasing MTR_asym_. Other studies employing BSA phantoms^21^ or *in vivo* 5, 10 all used much shorter (4 s) and higher powers (1.3–1.5 μT), which are less sensitive to the slowly exchanging NOE effects.

Other methods not examined in this study can also be used to quantify the various contributions to a measured CEST effect, such as qCEST.^14^ However, qCEST separates the contributions of labile proton exchange rate and concentration to the *Z* spectrum by measuring the CEST effect as a function of the saturation power. When applied to a clinical setting this approach is impractical owing to acquisition duration and specific absorption rate (SAR) concerns. In addition, the phantom model used in this study does not include a contribution from macromolecular magnetization transfer, which may remain as a confounding issue in interpreting changes in CEST metrics *in vivo*. However, macromolecular magnetization transfer effects can be accounted for by CESTR* with measured *T*_1_/*T*_2_ priors by including another pool in the BayCEST fitting algorithm.

### 4.1 Sensitivity of CESTR* to pH changes in tumours

One major result of this phantom study is that CESTR* measurement is dependent on a good choice of prior values of *T*_1_ and *T*_2_ time used by the BayCEST fitting algorithm. This implies that there is insufficient information in a single *Z* spectrum for the BayCEST algorithm to accurately estimate water *T*_1_ and *T*_2_ times when provided with generic, rather than individually measured, values for the prior distributions. The CESTR* metric with default *T*_1_/*T*_2_ priors has been used to identify the ischemic penumbra in acute stroke patients,^7^ and to generate quantitative pH maps in healthy volunteers and acute stroke patients.^3^ The apparent success of these pH maps (i.e. that the anticipated drop in pH was observed in known stroke regions) may be due to the lack of variation in *T*_1_ across the healthy brain and in the acute stage of stroke. In addition, the simulated CESTR*–pH calibration in that study was generated using a constant amide concentration of 100 mM.

However, assumptions of constant *T*_1_ and amide concentration may not be valid when assessing tumours. Consequently, accurate estimations of both *T*_1_ and labile proton concentration are necessary for reliable pH quantification in tumours using CESTR*. While *T*_1_ is easy to measure *in vivo*, the labile proton concentration is much harder to quantify reliably. We have shown in this phantom study that CESTR*, with knowledge of the *T*_1_ time and labile proton concentration, provides a means for immediate, quantitative and non‐invasive pH measurement, with an accuracy of 0.6 pH units based on the root mean square deviation of Figure 5. However, as the ΔpH measurements in Figure 5 were made on phantoms with known BSA concentration, application of this method *in vivo* is limited. Importantly, the non‐significant difference in sensitivities of CESTR* to pH in both tumour and normal brain phantoms implies that no manual tumour segmentation should be necessary when evaluating the pH of tumours using CESTR* *in vivo*.

Extracellular pH acidifications of the order of 0.6 pH units are not uncommon in tumours *in vivo*.^9^ Though intracellular pH changes of this order are rare, there are numerous cellular environments (mitochondria and other organelles) where the pH is very alkaline, and which may contribute to the CEST signal by virtue of the majority of their protein content being largely mobile.^29^, ^30^ The exact contribution of each of these compartments to the CEST signal measured *in vivo* remains to be elucidated.

## 5 CONCLUSION

Novel, realistic phantom models of the *in vivo* intracellular environment of brain metastases and naïve mouse brain have been developed to determine an analysis metric for quantification of CEST MRI data that is sensitive to only labile proton exchange rate and concentration. We demonstrate that the CESTR* metric with *T*_1_ and *T*_2_ time compensation overcomes many challenges facing interpretation of CEST MRI data. When combined with prior knowledge of protein concentration, CESTR* with *T*_1_/*T*_2_ compensation allows quantification of pH differences with a mean accuracy of 0.6 pH units. These results suggest that CEST MRI may enable pH differences between tumour and normal tissue to be quantified *in vivo* without the need for exogenous contrast agents.

## Supporting information


**Supplementary Figure S1:** Demonstration of the applicability of the Bayesian Bloch‐McConnell fitting algorithm BayCEST to measured *Z* spectra. The measured data are from an 8% *w*/*v* BSA supplemented with 4 T1‐GFP PCA extract phantom at pH 6.0 (left) and pH 7.6 (right). The red line shows the result of the Bloch‐McConnell equation fitting with three pools. The residual between the measured data and fitted Z‐spectrum is also shown, demonstrating a good fit for both phantoms, with the largest residuals appearing around the water frequency.
The insets in each panel show a zoomed version of the data and Bayesian Bloch‐McConnell fit for offset frequencies 1–5 ppm. Looking at the raw *Z* spectra, it is apparent that two peaks appear at higher pH. However, in this study, a 3 pool model fits the low and high pH phantoms equally well (pH = 6.0 data are fitted with R^2^ = 0.9849, pH = 7.6 data fitted with R^2^ = 0.9892). Visually, however, it appears that the fits could be improved by moving to a 4 pool model at high pH. We have chosen to maintain the 3 pool model because we would be in danger of over‐fitting the measured *Z* spectra, and the results of this phantom study would have limited applicability to the in vivo environment.
**Supplementary Figure S2:** Graphical representation of the CESTR* calculation procedure. The W and W + CEST *Z* spectra are simulated using the Bloch‐McConnell equations. The exchange rate and concentration parameters used in the simulation of W + CEST are those fitted from the Bayesian Bloch‐McConnell fitting (see Supplem[Link]), and the T_1_ and T_2_ times of each pool are kept constant. CESTR* is calculated as the difference in Z‐spectrum signal at the frequency of interest between the 1‐pool and 2‐pool simulations.
**Supplementary Figure S3:** The measured variation in T_2_ time for varying concentration of gadolinium‐DTPA to adjust T_1_ time (A) and T_1_ time for varying concentration of iron nanoparticles to adjust T_2_ time (B), for tumour (solid circles) and naïve brain (open squares) phantoms. Solid and dashed lines show the linear regression of both relationships for tumour and naïve brain phantoms, respectively. In all cases a significant variation in both relaxation times is measured, highlighting the difficulty in altering relaxation times independently with contrast agents. However, the variation in T_1_ with varying iron nanoparticle concentration to change T_2_ was four times less (76% change in T_2_ vs. 18% change in T_1_). Similarly, the variation in T_2_ as gadolinium‐DTPA was added to adjust T_1_ time was much less (78% change in T_1_ vs. 34% change in T_2_). Hence, the T_1_ and T_2_ times were treated as remaining constant for increasing concentration of iron nanoparticles and gadolinium‐DTPA, respectively, since the change in the target relaxation time was at least twice that of the other relaxation time.
**Supplementary Figure S4**: Raw Z‐spectrum for a tumour phantom with T_2_ relaxation time of 29 ms, showing that at such short T_2_ relaxation times the broadening of the water lineshape in the Z‐spectrum prevents the delineation of any discernible CEST peak at 2.8 ppm. This gives Z_ref_(2.8 ppm) < Z(2.8 ppm), and hence negative APT* and MTR_Rex_ values.
**Supplementary Figure S5:** Z‐spectrum (left) and MTRasym spectrum (right) acquired from a phantom containing only 3 M perchloric acid (PCA) used to extract the metabolites from 4 T1 cells and naïve brain tissue in this study. No CEST effect is discernible in the Z‐spectrum, indicating that no contaminant effect from the PCA is expected in our phantoms.
**Supplementary Figure S6:** Goodness‐of‐fit (R^2^) for a 3‐pool Bayesian Bloch‐McConnell fitting algorithm as shown in Supplem[Link] varies as the pH of the phantom changes. Goodness‐of‐fit is consistently high (> 0.976) for the full range of pH values.
**Supplementary Table 1:** Model parameter prior values used for Bayesian fitting of Bloch‐McConnell equations to measured Z‐spectra. Values are expressed as the mean ± standard deviation of a normal distribution.

Supporting info item
