# Supplementary material for: Determination of an optimally sensitive and specific chemical exchange saturation transfer MRI quantification metric in relevant biological phantoms
Source: NMR Biomed. 2016 Sep 30;29(11):1624–33. doi: 10.1002/nbm.3614 (PMC5095597; doi:10.1002/nbm.3614)
Supplement: Supplementary file 1 — Supporting info item [file NBM-29-1624-s001.docx]

**Determination of an optimally sensitive and specific Chemical Exchange Saturation Transfer MRI quantification metric in relevant biological phantoms**

Kevin J. Ray^1^, James R. Larkin^1^, Yee K. Tee^2^, Alexandre A. Khrapitchev^1^, Gogulan Karunanithy^3^, Michael Barber^3^, Andrew J. Baldwin^3^, Michael A. Chappell^4†^, Nicola R. Sibson ^1†^

*^1^Cancer Research UK and Medical Research Council Oxford Institute for Radiation Oncology, Department of Oncology, University of Oxford, Oxford OX3 7LE, UK*

*^2^Department of Mechatronics and Biomedical Engineering, Lee Kong Chian Faculty of Engineering and Science, Universiti Tunku Abdul Rahman, Malaysia.*

*^3^Physical and Theoretical Chemistry, University of Oxford, Oxford, OX1 3QZ, UK.*

*^4^Institute for Biomedical Engineering, University of Oxford, Oxford, OX1 7LE, UK.*

† Joint senior authors

**SUPPLEMENTARY FIGURES AND TABLES**

|  | **Water Pool** | **CEST Pool** | **NOE Pool** |
| --- | --- | --- | --- |
| **Offset Frequency (ppm)** | 0 | 2.8 ± 0.3 | -3.5 ± 0.3 |
| **k_i,w_(Hz)** | - | 500 | 50 |
| **log(k_i,w_)*** | - | 2.7 ± 1.0 | 1.7 ± 1.0 |
| **M^i^_0_** | 0 ± 10^6^ | - | - |
| **rM^i^_0_ (M^i^_0_ / M^w^_0_)** | - | 0.0 ± 0.1 | 0.0 ± 0.1 |
| **T_1_ (s)^#^** | 1.80 ± 0.36  or  From T_1_ Map | 1.80 ± 0.36 | 1.80 ± 0.36 |
| **T_2_ (ms)^#^** | 100 ± 20  or  From T_2_ Map | 0.25 ± 0.05 | 0.25 ± 0.05 |
| **Supplementary Table 1:** Model parameter prior values used for Bayesian fitting of Bloch-McConnell equations to measured Z-spectra. Values are expressed as the mean ± standard deviation of a normal distribution.  * indicates that the log value of the exchange rate was fitted during the model fitting, so the prior and standard deviation were defined as the log value.  # indicates that the mean and standard deviation defined for the T_1_ time or T_2_ time was varied to either be identical for all phantoms or varied each time to be the values measured by the T_1_ and T_2_ maps. | | | |


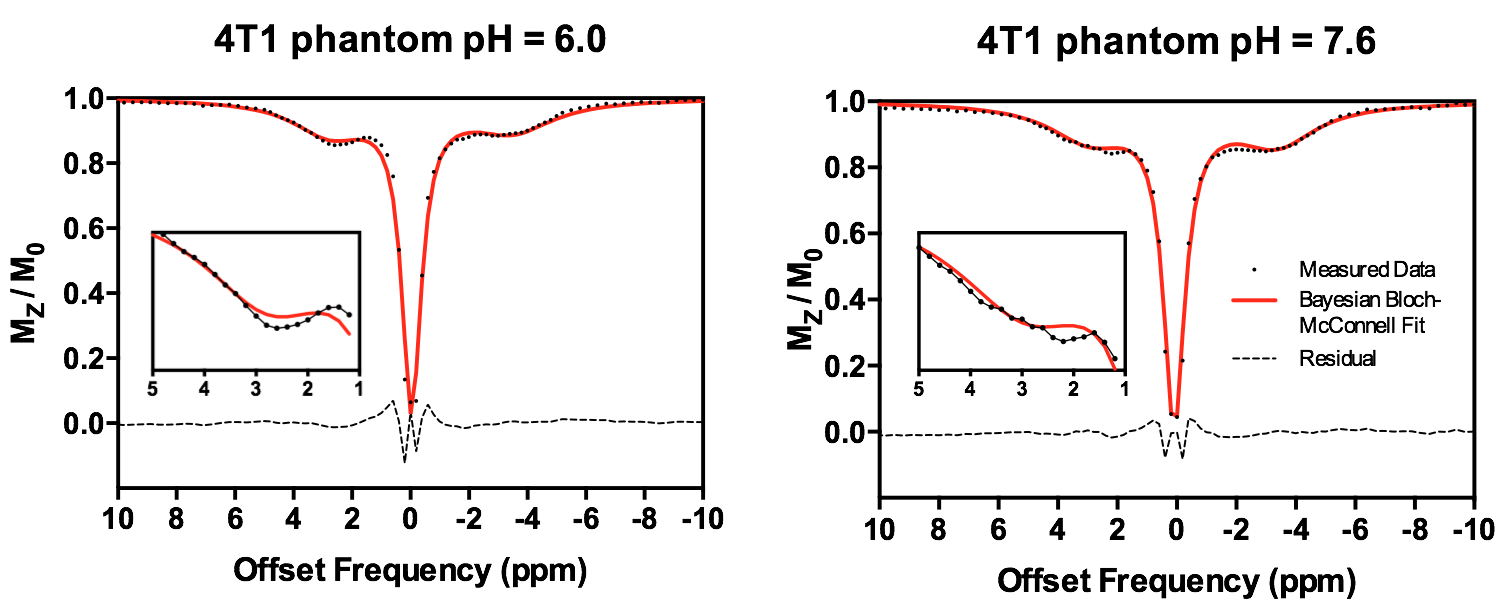


**Supplementary Figure S1:** Demonstration of the applicability of the Bayesian Bloch-McConnell fitting algorithm BayCEST to measured Z-spectra. The measured data are from an 8% w/v BSA supplemented with 4T1-GFP PCA extract phantom at pH 6.0 (left) and pH 7.6 (right). The red line shows the result of the Bloch-McConnell equation fitting with three pools. The residual between the measured data and fitted Z-spectrum is also shown, demonstrating a good fit for both phantoms, with the largest residuals appearing around the water frequency.

The insets in each panel show a zoomed version of the data and Bayesian Bloch-McConnell fit for offset frequencies 1 – 5 ppm. Looking at the raw Z-spectra, it is apparent that two peaks appear at higher pH. However, in this study, a 3 pool model fits the low and high pH phantoms equally well (pH = 6.0 data are fitted with R^2^ = 0.9849, pH = 7.6 data fitted with R^2^ = 0.9892). Visually, however, it appears that the fits could be improved by moving to a 4 pool model at high pH. We have chosen to maintain the 3 pool model because we would be in danger of over-fitting the measured Z-spectra, and the results of this phantom study would have limited applicability to the in vivo environment.


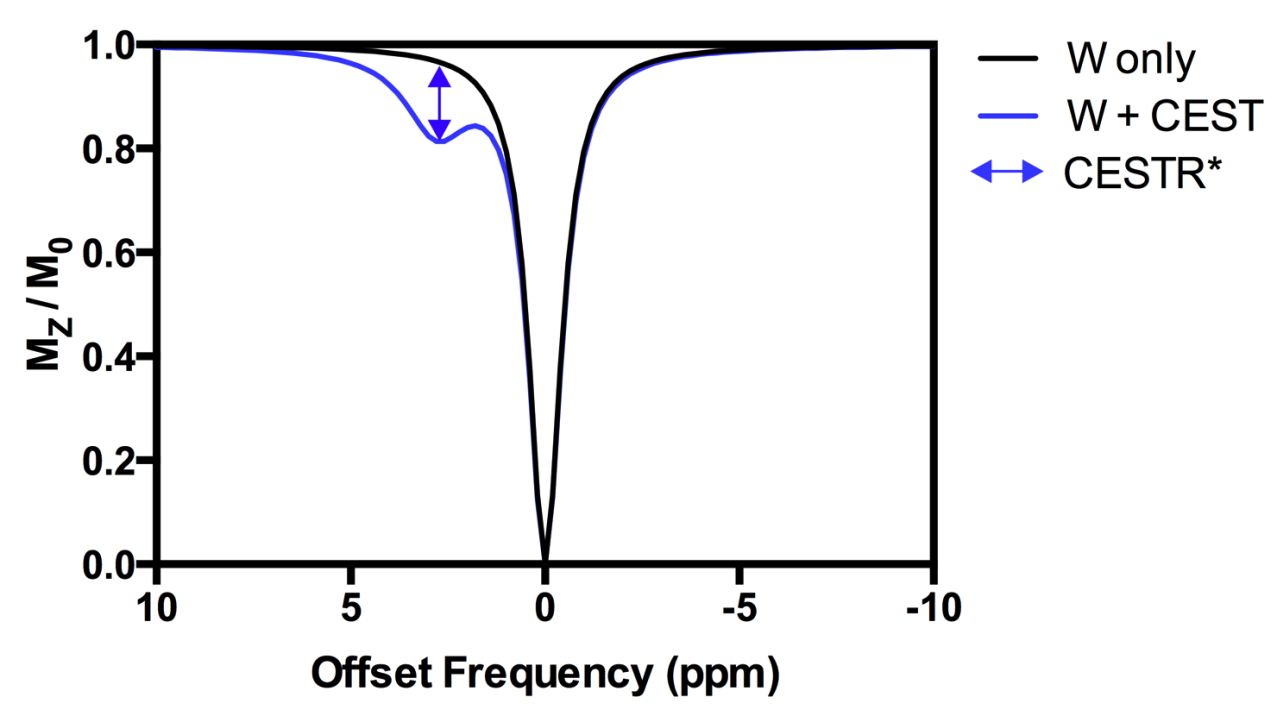


**Supplementary Figure S2:** Graphical representation of the CESTR* calculation procedure. The W and W + CEST Z-spectra are simulated using the Bloch-McConnell equations. The exchange rate and concentration parameters used in the simulation of W + CEST are those fitted from the Bayesian Bloch-McConnell fitting (see Supplementary Figure 1), and the T_1_ and T_2_ times of each pool are kept constant. CESTR* is calculated as the difference in Z-spectrum signal at the frequency of interest between the 1-pool and 2-pool simulations.


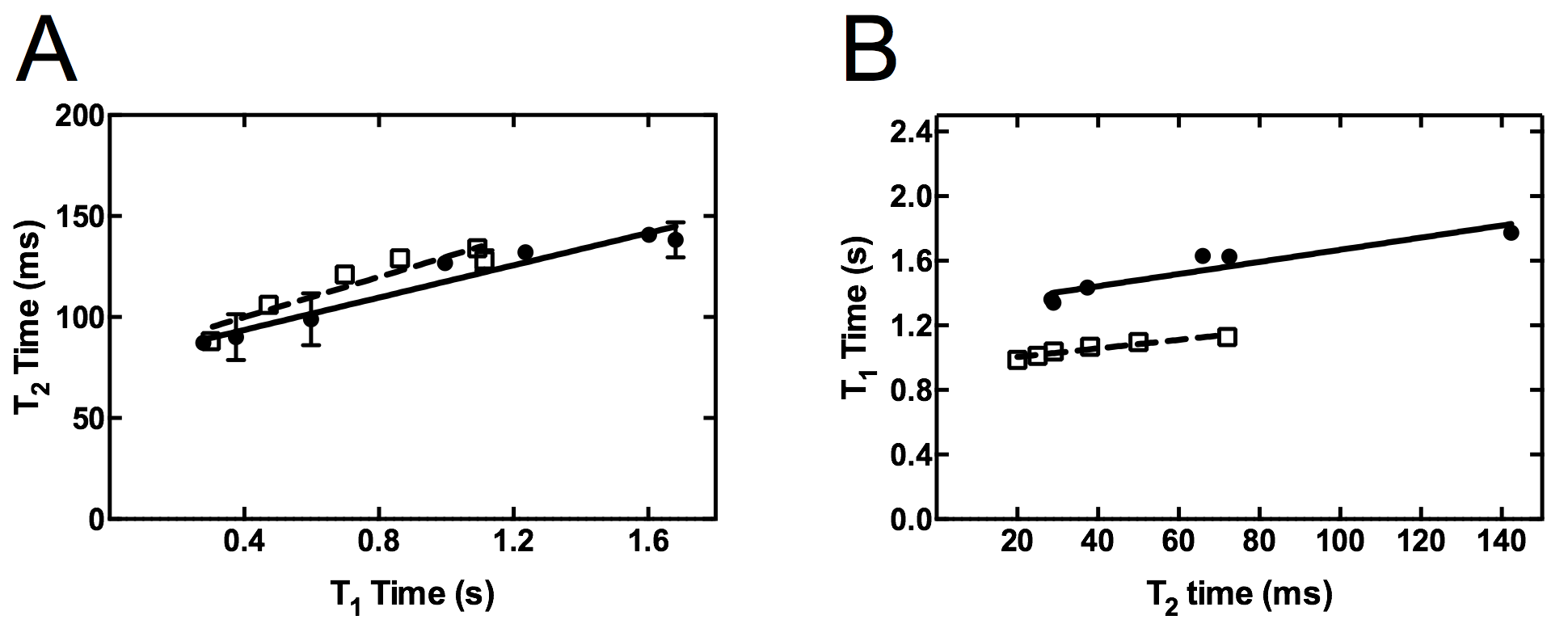


**Supplementary Figure S3:** The measured variation in T_2_ time for varying concentration of gadolinium-DTPA to adjust T_1_ time (A) and T_1_ time for varying concentration of iron nanoparticles to adjust T_2_ time (B), for tumour (solid circles) and naïve brain (open squares) phantoms. Solid and dashed lines show the linear regression of both relationships for tumour and naïve brain phantoms, respectively. In all cases a significant variation in both relaxation times is measured, highlighting the difficulty in altering relaxation times independently with contrast agents. However, the variation in T_1_ with varying iron nanoparticle concentration to change T_2_ was four times less (76% change in T_2_ vs. 18% change in T_1_). Similarly, the variation in T_2_ as gadolinium-DTPA was added to adjust T_1_ time was much less (78% change in T_1_ vs. 34% change in T_2_). Hence, the T_1_ and T_2_ times were treated as remaining constant for increasing concentration of iron nanoparticles and gadolinium-DTPA, respectively, since the change in the target relaxation time was at least twice that of the other relaxation time.

**Supporting Figure S4**: Raw Z-spectrum for a tumour phantom with T_2_ relaxation time of 29 ms, showing that at such short T_2_ relaxation times the broadening of the water lineshape in the Z-spectrum prevents the delineation of any discernible CEST peak at 2.8ppm. This gives Z_ref_(2.8ppm) < Z(2.8ppm), and hence negative APT* and MTR_Rex_ values.


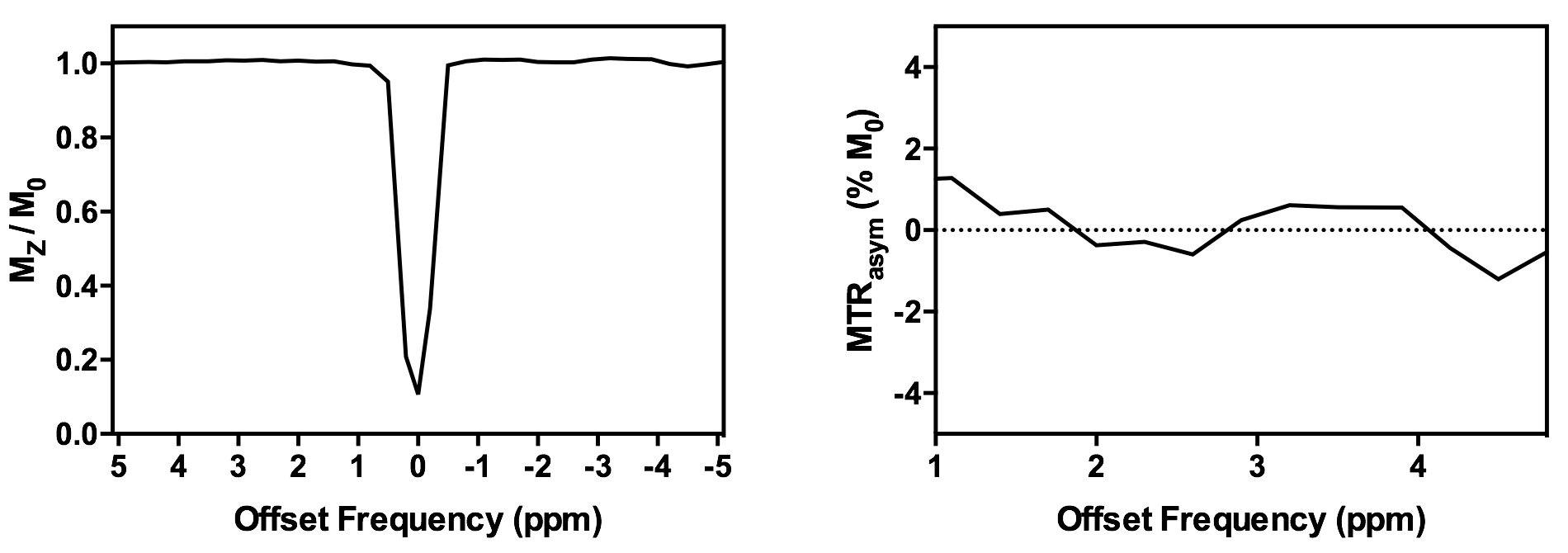


**Supplementary Figure S5:** Z-spectrum (left) and MTR_asym_ spectrum (right) acquired from a phantom containing only 3M perchloric acid (PCA) used to extract the metabolites from 4T1 cells and naïve brain tissue in this study. No CEST effect is discernible in the Z-spectrum, indicating that no contaminant effect from the PCA is expected in our phantoms.


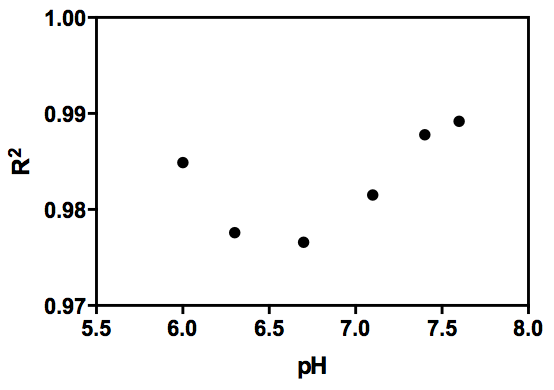


**Supplementary Figure S6:** Goodness-of-fit (R^2^) for a 3-pool Bayesian Bloch-McConnell fitting algorithm as shown in Supplementary Figure S1 varies as the pH of the phantom changes. Goodness-of-fit is consistently high (> 0.976) for the full range of pH values.
